# Supplementary material for: Distinct sensorimotor feedback loops for dynamic and static control of primate precision grip
Source: Commun Biol. 2020 Apr 2;3:156. doi: 10.1038/s42003-020-0861-0 (PMC7118171; doi:10.1038/s42003-020-0861-0)
Supplement: Supplementary file 1 — Supplementary Information [file 42003_2020_861_MOESM1_ESM.pdf]

Supplementary Figures

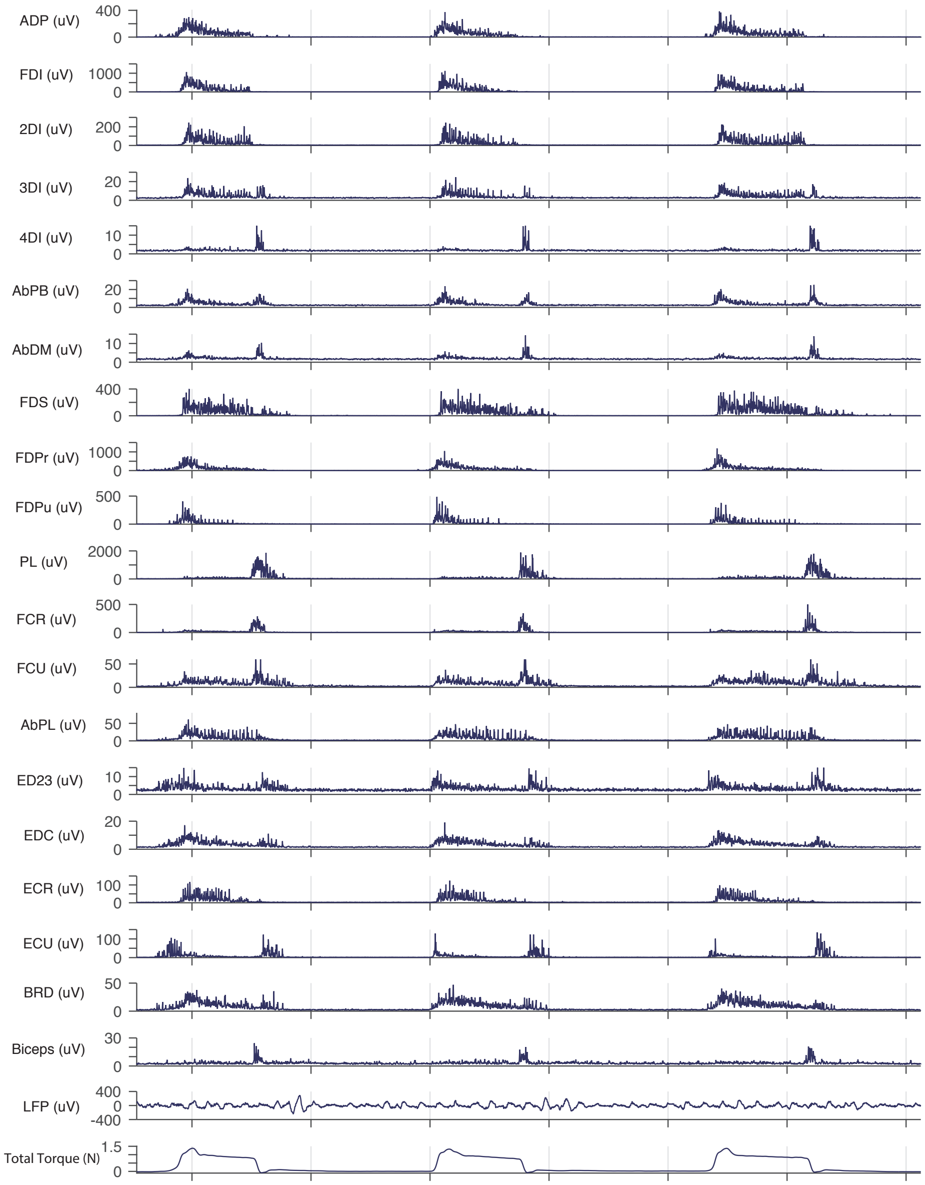

Supplementary Figure 1. Example traces for simultaneously recorded EMGs, LFPs (spinal cord), and finger force from a monkey performing a precision grip task.

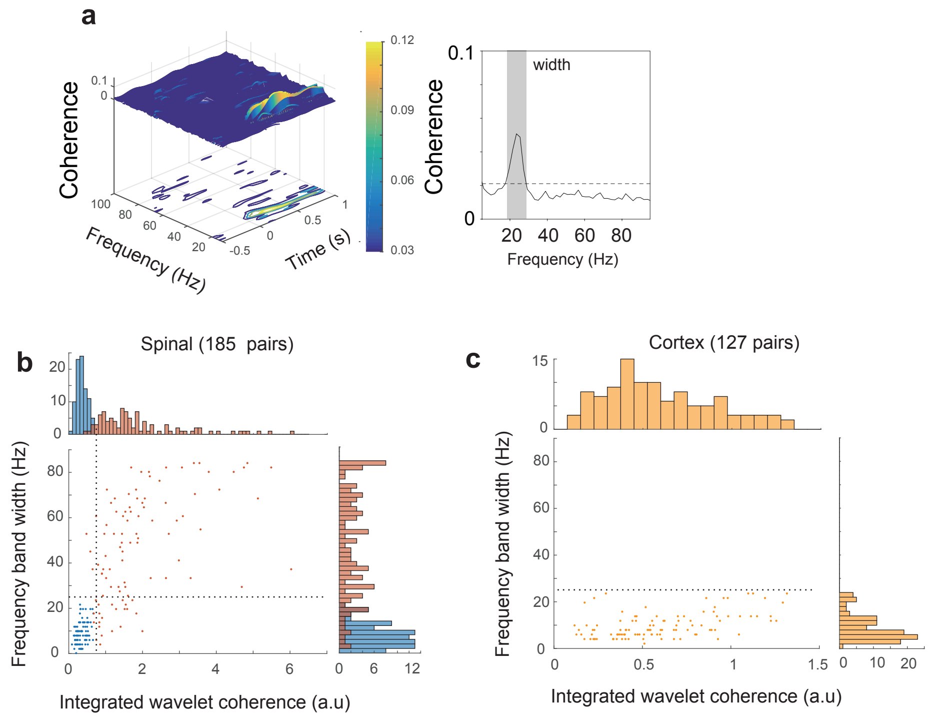

Supplementary Figure 2 Group classification of coherence patterns. **a** Integrated contour in wavelet coherence and the frequency width in standard coherence for classification. The integrated contour was calculated as a sum of the area above the significance level from the wavelet coherence, whereas the frequency width was taken as consecutive coherence above the significance level at its highest peak (shaded area). **b, c** Scatterplots and marginal distributions of the coherence width against the integrated contour, for spinomuscular coherence (**b**) and corticomuscular coherence (**c**).

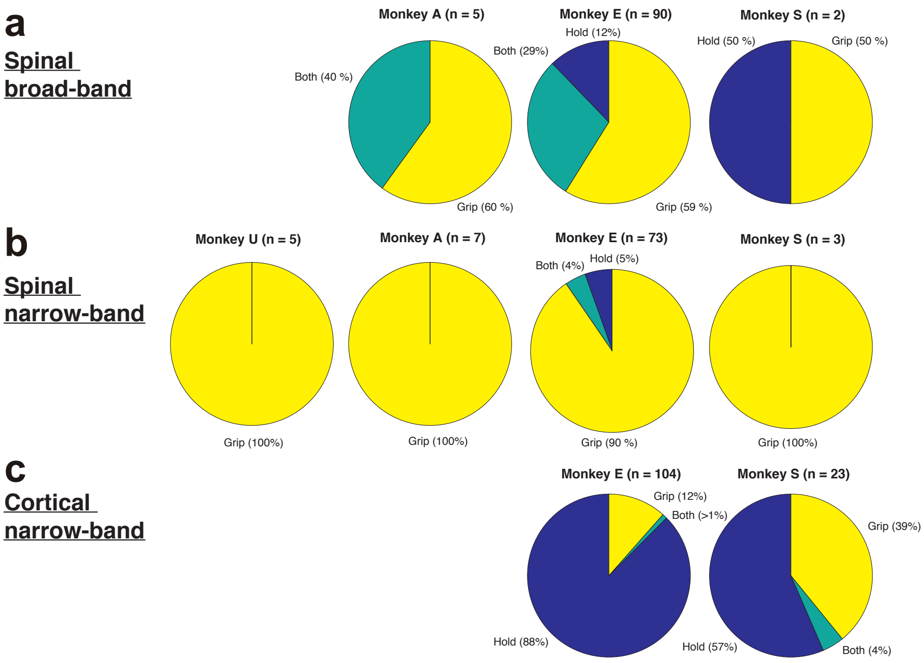

Supplementary Figure 3. Pie charts showing proportions of significant coherence found in grip-only, hold-only, and both for spinal narrow-band (**a**), broad-band (**b**), and cortical narrow-band (**c**) coherence in individual animals.

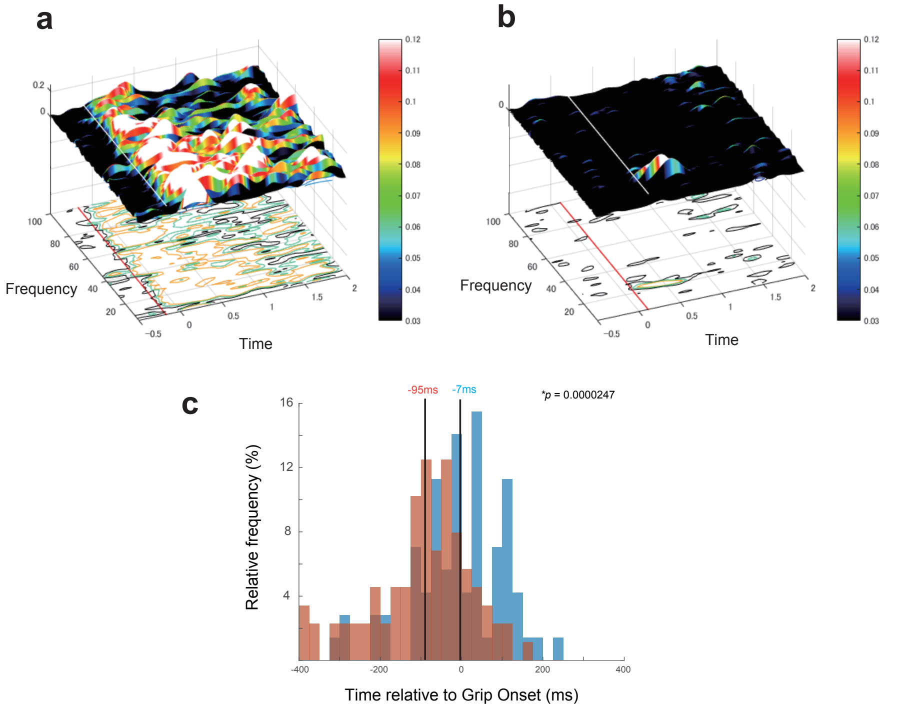

Supplementary Figure 4. Comparison of latencies for spinal BB and NB coherence with respect to grip onset. **a, b** Representative spectrograms and contours for calculating the onsets for spinal BB and for NB. White lines in spectrograms and red lines in contours stand for the onsets of the coherence, calculated as medians of the first time that rises above significant levels (black contours) in all significant bands. **c** Group histograms for spinal BB and NB coherence during the grip. The median latency for BB (red bars: -95 ms) was significantly earlier than that for NB (blue bars: -7 ms). The *p*-value was calculated using *t*-test with unequal variance.

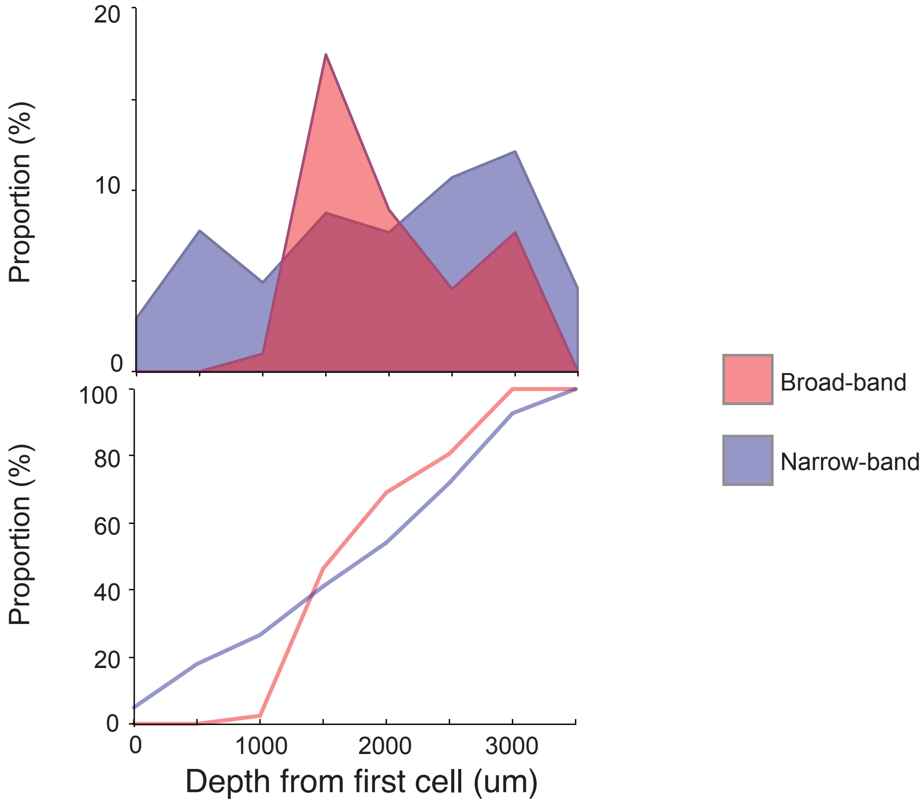

Supplementary Figure 5. Distribution of spinal BB and NB coherence according to the depth of recording sites. The depth of a recording site is expressed relative to the depth where the first unit activity was encountered in each penetration. The distribution (upper panel) of BB (red) and NB (blue) pairs, as expressed as proportions with respect to total sites found for each categories (55 for BB and 68 for NB), and their cumulative distributions (lower panel) are shown. While the NB coherence was observed throughout the depth, BB coherence was found at specific depths ($p = 0.0034$, χ^2 test).

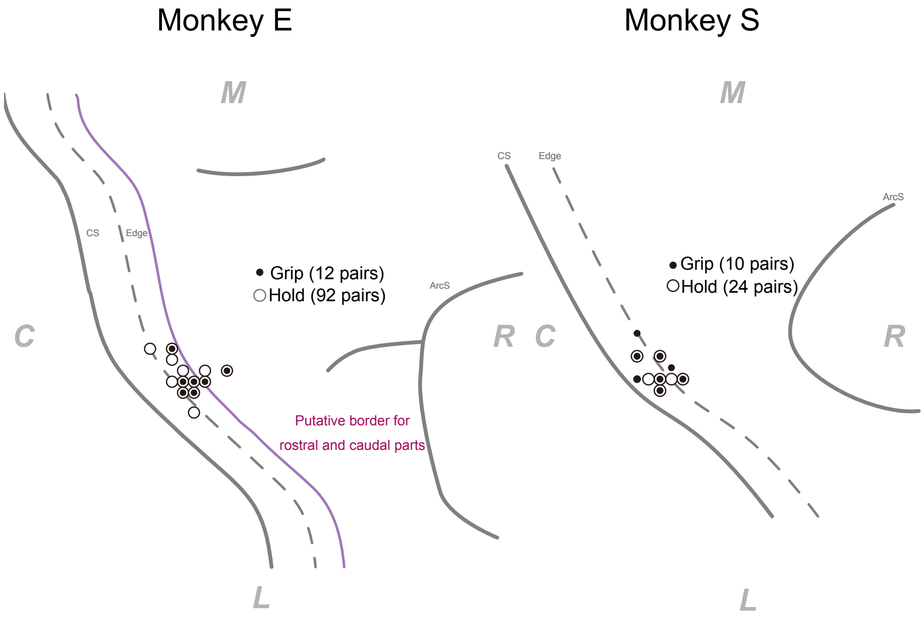

Supplementary Figure 6. Surface maps of recording sites in the motor cortex, from monkey E and S. Each filled or open circle represents the track where at least one pair of LFP-EMG coherence was observed during either grip or hold phase. Total numbers of pairs of coherence are described next to the legend. R for rostral, C for caudal, M for medial, and L for lateral directions, respectively. The putative border for rostral and caudal subdivisions were defined based on the intracortical microstimulation [1].

Supplementary References

[1] Crammond, D. J. & Kalaska, J. F. Differential relation of discharge in primary motor cortex and premotor cortex to movements versus actively maintained postures during a reaching task. *Exp. Brain. Res.* **108**, 45–61 (1996).
