# Supplementary material for: Distinct sensorimotor feedback loops for dynamic and static control of primate precision grip
Source: Commun Biol. 2020 Apr 2;3:156. doi: 10.1038/s42003-020-0861-0 (PMC7118171; doi:10.1038/s42003-020-0861-0)
Supplement: Supplementary file 2 — Description of Additional Supplementary Files [file 42003_2020_861_MOESM2_ESM.pdf]

Description of Additional Supplementary Files

File Name: Supplementary Data 1
Description: All source data supporting the figures shown in the main text.
