# Supplementary material for: Distinct sensorimotor feedback loops for dynamic and static control of primate precision grip
Source: Commun Biol. 2020 Apr 2;3:156. doi: 10.1038/s42003-020-0861-0 (PMC7118171; doi:10.1038/s42003-020-0861-0)
Supplement: Supplementary file 4 — Reporting Summary [file 42003_2020_861_MOESM4_ESM.pdf]

nature research

Corresponding author(s): Kazuhiko Seki

Last updated by author(s): Feb 7, 2020

Reporting Summary

Nature Research wishes to improve the reproducibility of the work that we publish. This form provides structure for consistency and transparency in reporting. For further information on Nature Research policies, see Authors & Referees and the Editorial Policy Checklist .

Statistics

For all statistical analyses, confirm that the following items are present in the figure legend, table legend, main text, or Methods section.

n/a	Confirmed	
☐	☒	The exact sample size (*n*) for each experimental group/condition, given as a discrete number and unit of measurement
☐	☒	A statement on whether measurements were taken from distinct samples or whether the same sample was measured repeatedly
☐	☒	The statistical test(s) used AND whether they are one- or two-sided<br>*Only common tests should be described solely by name; describe more complex techniques in the Methods section.*
☒	☐	A description of all covariates tested
☒	☐	A description of any assumptions or corrections, such as tests of normality and adjustment for multiple comparisons
☐	☒	A full description of the statistical parameters including central tendency (e.g. means) or other basic estimates (e.g. regression coefficient) AND variation (e.g. standard deviation) or associated estimates of uncertainty (e.g. confidence intervals)
☐	☒	For null hypothesis testing, the test statistic (e.g. *F*, *t*, *r*) with confidence intervals, effect sizes, degrees of freedom and *P* value noted<br>*Give P values as exact values whenever suitable.*
☒	☐	For Bayesian analysis, information on the choice of priors and Markov chain Monte Carlo settings
☒	☐	For hierarchical and complex designs, identification of the appropriate level for tests and full reporting of outcomes
☒	☐	Estimates of effect sizes (e.g. Cohen's *d*, Pearson's *r*), indicating how they were calculated

Our web collection on statistics for biologists contains articles on many of the points above.

Software and code

Policy information about availability of computer code

Data collection	Data collection was made using MSD software (Alpha Omega Engineering, Nazareth, Israel).
Data analysis	Custom written MATLAB (R2010b, R2016b) routines were used for data analyses. For Multivariate autoregressive model analyses (MVAR), we used ARFit packages (Schneider and Neumaier, 2001, publicly available at https://jp.mathworks.com/matlabcentral/fileexchange/174-arfit, or https://github.com/tapios/arfit) to write the routines. For circular statistics on phase distributions, we used CircStat Toolbox (https://www.jstatsoft.org/article/view/v031i10).

For manuscripts utilizing custom algorithms or software that are central to the research but not yet described in published literature, software must be made available to editors/reviewers. We strongly encourage code deposition in a community repository (e.g. GitHub). See the Nature Research guidelines for submitting code & software for further information.

Data

Policy information about availability of data

All manuscripts must include a data availability statement. This statement should provide the following information, where applicable:

- Accession codes, unique identifiers, or web links for publicly available datasets
- A list of figures that have associated raw data
- A description of any restrictions on data availability

The datasets that support the findings of the current study are available from the corresponding author upon reasonable request.

Field-specific reporting

Please select the one below that is the best fit for your research. If you are not sure, read the appropriate sections before making your selection.

Life sciences study design

All studies must disclose on these points even when the disclosure is negative.

Sample size	No statistical methods were used to pre-determine sample sizes. Our sample sizes were determined on basis of the previous reports using similar experimental procedures.
Data exclusions	We excluded EMG data with electrical cross-talks that could influence the coherence analyses. The details are referred to Methods section. Also, to eliminate any influence from the power line, we excluded the frequency band around 50 or 60 Hz, and concatenated the neighboring frequencies.
Replication	Replication of the results was confirmed in four monkeys.
Randomization	Since no experimental groups were assigned, randomization is not relevant to this study.
Blinding	Since no group allocation was performed for the data collection or analyses, blinding is not relevant to this study.

Reporting for specific materials, systems and methods

We require information from authors about some types of materials, experimental systems and methods used in many studies. Here, indicate whether each material, system or method listed is relevant to your study. If you are not sure if a list item applies to your research, read the appropriate section before selecting a response.

Materials & experimental systems

n/a	Involved in the study	
☒	☐	Antibodies
☒	☐	Eukaryotic cell lines
☒	☐	Palaeontology
☐	☒	Animals and other organisms
☒	☐	Human research participants
☒	☐	Clinical data

Methods

n/a	Involved in the study	
☒	☐	ChIP-seq
☒	☐	Flow cytometry
☒	☐	MRI-based neuroimaging

Animals and other organisms

Policy information about studies involving animals; ARRIVE guidelines recommended for reporting animal research

Laboratory animals	The datasets used in the present study were obtained from four male macaque monkeys consisting of three Japanese monkeys (Macaca fuscata; monkey U: 8.5 kg, 7 years old, monkey A: 6.8 kg 6 years old, monkey S: 9.0 kg, 8 years old, at the time of data collection) and one rhesus monkey (Macaca mulatta; monkey E: 5.6 kg, 5 years old at the time of data collection).
Wild animals	No wild animals were used in this study.
Field-collected samples	No field-collected samples were used in this study.
Ethics oversight	All surgical and experimental procedures were approved by Animal Research Committees at National Institute for Physiological Sciences, and National Center of Neurology and Psychiatry, Japan.

Note that full information on the approval of the study protocol must also be provided in the manuscript.
